# Moisture-induced solid state instabilities in α-chymotrypsin and their reduction through chemical glycosylation

**DOI:** 10.1186/1472-6750-10-57

**Published:** 2010-08-09

**Authors:** Giselle M Flores-Fernández, Miraida Pagán, Mariangely Almenas, Ricardo J Solá, Kai Griebenow

**Affiliations:** 1 Department of Chemistry, University of Puerto Rico, Río Piedras Campus, P.O. Box 23346, San Juan, 00931-3346, Puerto Rico

## Abstract

**Background:**

Protein instability remains the main factor limiting the development of protein therapeutics. The fragile nature (structurally and chemically) of proteins makes them susceptible to detrimental events during processing, storage, and delivery. To overcome this, proteins are often formulated in the solid-state which combines superior stability properties with reduced operational costs. Nevertheless, solid protein pharmaceuticals can also suffer from instability problems due to moisture sorption. Chemical protein glycosylation has evolved into an important tool to overcome several instability issues associated with proteins. Herein, we employed chemical glycosylation to stabilize a solid-state protein formulation against moisture-induced deterioration in the lyophilized state.

**Results:**

First, we investigated the consequences of moisture sorption on the stability and structural conformation of the model enzyme α-chymotrypsin (α-CT) under controlled humidity conditions. Results showed that α-CT aggregates and inactivates as a function of increased relative humidity (RH). Furthermore, α-CT loses its native secondary and tertiary structure rapidly at increasing RH. In addition, H/D exchange studies revealed that α-CT structural dynamics increased at increasing RH. The magnitude of the structural changes in tendency parallels the solid-state instability data (i.e., formation of buffer-insoluble aggregates, inactivation, and loss of native conformation upon reconstitution). To determine if these moisture-induced instability issues could be ameliorated by chemical glycosylation we proceeded to modify our model protein with chemically activated glycans of differing lengths (lactose and dextran (10 kDa)). The various glycoconjugates showed a marked decrease in aggregation and an increase in residual activity after incubation. These stabilization effects were found to be independent of the glycan size.

**Conclusion:**

Water sorption leads to aggregation, inactivation, and structural changes of α-CT as has been similarly shown to occur for many other proteins. These instabilities correlate with an increase in protein structural dynamics as a result of moisture exposure. In this work, we present a novel methodology to stabilize proteins against structural perturbations in the solid-state since chemical glycosylation was effective in decreasing and/or preventing the traditionally observed moisture-induced aggregation and inactivation. It is suggested that the stabilization provided by these chemically attached glycans comes from the steric hindrance that the sugars conveys on the protein surface therefore preventing the interaction of the protein internal electrostatics with that of the water molecules and thus reducing the protein structural dynamics upon moisture exposure.

## Background

Many biopharmaceutical drugs, such as peptides and proteins, are currently in or close to entering clinical trials [1-5]. They hold promise in being effective in the prevention and cure of numerous diseases. Unfortunately, protein therapeutics display many stability problems, such as inactivation and aggregation during manufacturing and storage [2-6]. Of particular interest is the solid-state stability of proteins since lyophilization is frequently employed to enhanced the product shell-life and offers superior strategies to formulate inhalation drugs and delivery systems [6]. However, exposure of lyophilized proteins to excess moisture causes undesirable physical and chemical changes [1,3,7]. The residual water bound at the surface to charged and polar groups and to the peptide backbone groups can cause the protein to be unstable and degrade [8,9]. These drawbacks highlight the need for the development of rational formulation strategies to improve overall protein stability within multiple applications [2].

Profound changes are observed in protein structure and conformational dynamics depending on the hydration level that directly affect solid-phase stability [10,11]. It is commonly assumed that the hydration of solid proteins leads to increased rates of deleterious processes due to increased structural dynamics and higher mobility of reactive species [10,11]. Upon storage, proteins may absorb moisture and detrimental moisture- induced aggregation is observed. More importantly, there are also applications in which proteins are partially hydrated, for example in sustained release applications when lyophilized proteins are embedded in a polymer matrix and are slowly hydrated upon polymer swelling and erosion during release. (Note that data presented in this paper after incubation of the model protein at high moisture levels mostly refer to this scenario as it is unlikely that such substantial moisture adsorption can occur accidentally simply by opening a vial containing the lyophilized powder.)

The extent of protein aggregation has been related to the amount of water sorbed by the protein [10] and to residual water bound to charged and polar protein surface groups and the peptide backbone [9,12]. These aggregates cause lower activity and increased immunogenicity [1,10,13,14].

Many strategies are available to increase the long-term stability for solid-state protein formulations such as, co-dissolving additives or introducing mutations. Amongst them covalent chemical modification represents one of the most promising approaches for the stabilization of proteins in industrial and pharmaceutical applications [13,15]. In his context, chemical glycosylation has been used to increase physical protein stability [4,5,16-25]. Glycosylation consists in the modification of one or more protein surface residues (typically lysines) with chemically activated glycans, which leads to chemically stable conjugates that can be stored lyophilized [21]. Solá *et al. *demonstrated that increasing the size and amount of chemically attached glycans did not alter the original structural fold of the model protein α-chymotrypsin (αCT) employed and that a substantial decrease in protein structural dynamics was induced by glycosylation and as a result increased thermodynamic and colloidal stability was observed [21-24]. Similar data were obtained for the structurally unrelated serine protease subtilisin Carlsberg [25] supporting the view that the phenomenon of protein stabilization by chemical glycosylation is likely of a general nature [4,5,21]. Building up on those data in the present study we further employed α-CT as model enzyme. The studies performed in this paper consume rather large amounts of protein and a first study is probably best performed using an inexpensive model enzyme rather than an expensive protein drug or drug candidate. However, chymotrypsin also has a wide range of applications as biocatalyst and is used or has been suggested as therapeutic agent in anti-inflammatory treatment by injection, for cleaning of nectrotic wounds, and even as anti-cancer drug [26-28]. Anaphylactic shock has been reported after intramuscular injection of lyophilized α-chymotrypsin [29]. Since protein aggregates are highly suspicious in this context [14], strategies avoiding aggregation of α-chymotrypsin are necessary.

## Results and Discussion

### Moisture-induced Aggregation

α-CT was chosen as the model protein since it has been employed prior to this investigation by us in solid-phase stability studies and also as model protein to test drug- delivery systems [1,30,31]. α-CT is prone to undergo aggregation and thus is an excellent sensor for aggregation-related instability events. Our experimental approach was to expose solid α-CT formulations to accelerated storage conditions (i.e., high temperature) under controlled humidity conditions as it is common in the field [7,10,32]. We then monitored various stability related parameters as a function of time, i.e., loss in monomer concentration (by SEC-HPLC), residual specific activity of soluble α-CT, formation of buffer-insoluble aggregates, tertiary structure intactness of soluble α-CT (by CD spectroscopy), and structural dynamics (by FTIR H/D exchange).

**Figure 1:**
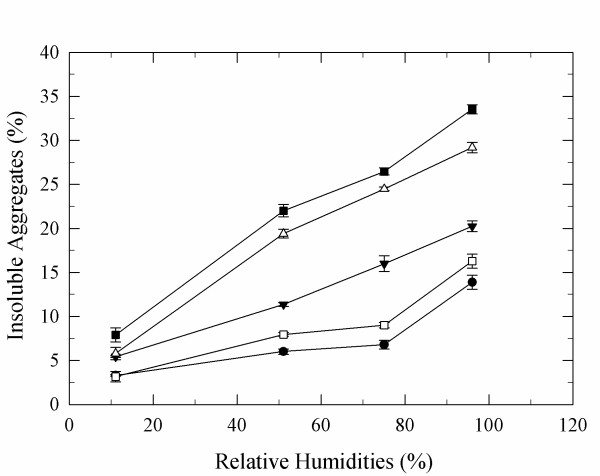
**Aggregation of lyophilized α-CT at 50°C and different RH**. α-CT was dissolved at pH 7.3 and the concentration prior to lyophilization was 0.5 mg/mL. 24 h (solid circle), 48 h (open square), 96 h (open down triangle), 1 week (open triangle), and 2 weeks (solid square).

To investigate the aggregation of α-CT at different RH, formation of insoluble aggregates was measured by solution-depletion experiments after incubation at 50°C and at 11%, 51%, 75%, 81%, and 96% RH for up to 3 weeks. Water-sorption isotherms demonstrated that all samples were completely equilibrated after 12 h (Additional file 1). All α-CT samples exhibited significant solubility loss during incubation due to aggregation (Figure 1). In contrast, α-CT stored at -20°C remained completely soluble in buffer. A control stored at 0% RH and 50°C did not form more than 2.1% aggregates. Thus, the detrimental process occurred during the high temperature/humidity incubation of solid α-CT, and not during lyophilization. Long-term storage of the protein at increasing levels of RH increased the formation of aggregates with this effect also increasing with storage time (Figure 1). Its aqueous solubility plummeted in particular after various days of incubation at 96% RH.

More than 90% of the aggregates were dissolved in 6 M urea which indicates that the α-CT molecules in the aggregates were held together by non-covalent interactions.

The Kruskall-Wallis test was employed to statistically investigate whether aggregate formation was significantly different at the various RH employed. The analysis revealed that there are marked differences in the amount of aggregates formed at the various RH employed (p < 0.05). To compare the effect of the RH on aggregate formation quantitatively, we performed a post hoc test (Dunn's test). This test revealed that aggregation was markedly different at RH of 11% and 51% RH when compared to 75%, 81%, and 96% RH and at 75% RH when compared to 96% RH. Furthermore, statistical analysis revealed that there was a significant difference in the formation of aggregates at various incubation times at each RH. Aggregation was markedly different after 24 h of incubation when compared to the samples stored for 1 and 2 weeks at RH of 51%, 75%, 81%, and 96%.

**Figure 2:**
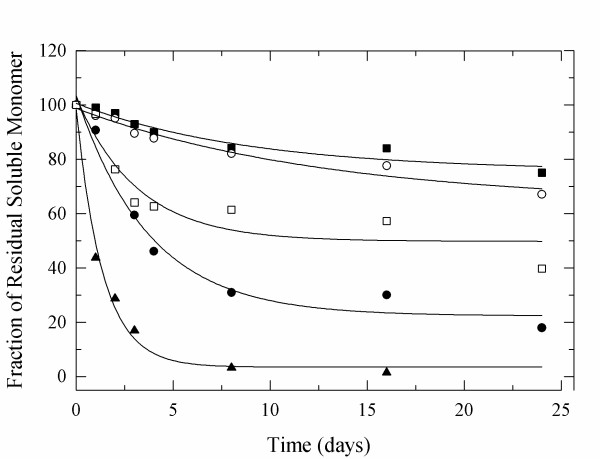
**Time course of moisture-induced aggregation of α-CT lyophilized from pH 5 as determined by SEC-HPLC**. 11% RH (solid squre), 51% RH (open circle), 75% RH (open square), 81% RH (solid circle), and 96% RH (solid triangle).

To study the kinetics of α-CT monomer loss, we investigated the water soluble fraction after dissolving the solid samples in buffer by SEC-HPLC. The amount of monomeric α-CT was measured based on the area of the corresponding HPLC peak (see Supporting Information for details) and the fraction of residual soluble monomer was plotted as a function of time (Figure 2). Two degradation pathways were identified: formation of soluble aggregates and fragmentation of the enzyme into subunits [22]. The kinetics of the monomer loss can be modeled using the following equation:

(1) $$\text{X}={\text{A}}_{1}\exp^{\left(-{\text{k}}_{1}t\right)}+{\text{A}}_{2}$$

where X is the fraction of residual soluble monomer and A1 represents the population of protein undergoing aggregation or fragmentation with the rate k1, and A2 is the population that remains unaffected.

The data obtained reveal that monomer loss was not as pronounced at 11% and 51% RH as at higher RH (Table 1, Figure 2). Between 51% and 95% RH monomer loss increased at increasing RH level. The differences observed when compared to formation of buffer-insoluble aggregates at the various RH were more pronounced. This can be understood because more than one off-pathway leads to monomer loss and in particular at high RH values fragmentation becomes a significant degradation pathway.

**Table 1:** Kinetic parameters determined from the monomer loss detected by SEC-HPLC of α-CT after incubation at different RH.

RH	**A**_**2**_	**A**_**1**_	**k**_**1**_
11%	75 ± 4	25 ± 4	0.122 ± 0.004
51%	64 ± 5	36 ± 4	0.078 ± 0.002
75%	50 ± 3	50 ± 5	0.317 ± 0.006
81%	22 ± 2	78 ± 3	0.267 ± 0.002
96%	3 ± 1	97 ± 2	0.736 ± 0.004

In general, the instability data parallel the water-sorption at the various RH (see Additional file 2 for details). Water sorption becomes significant at 51% RH and then steadily increases.

### Effect of Moisture on the Tertiary Structure of α-Chymotrypsin

**Figure 3:**
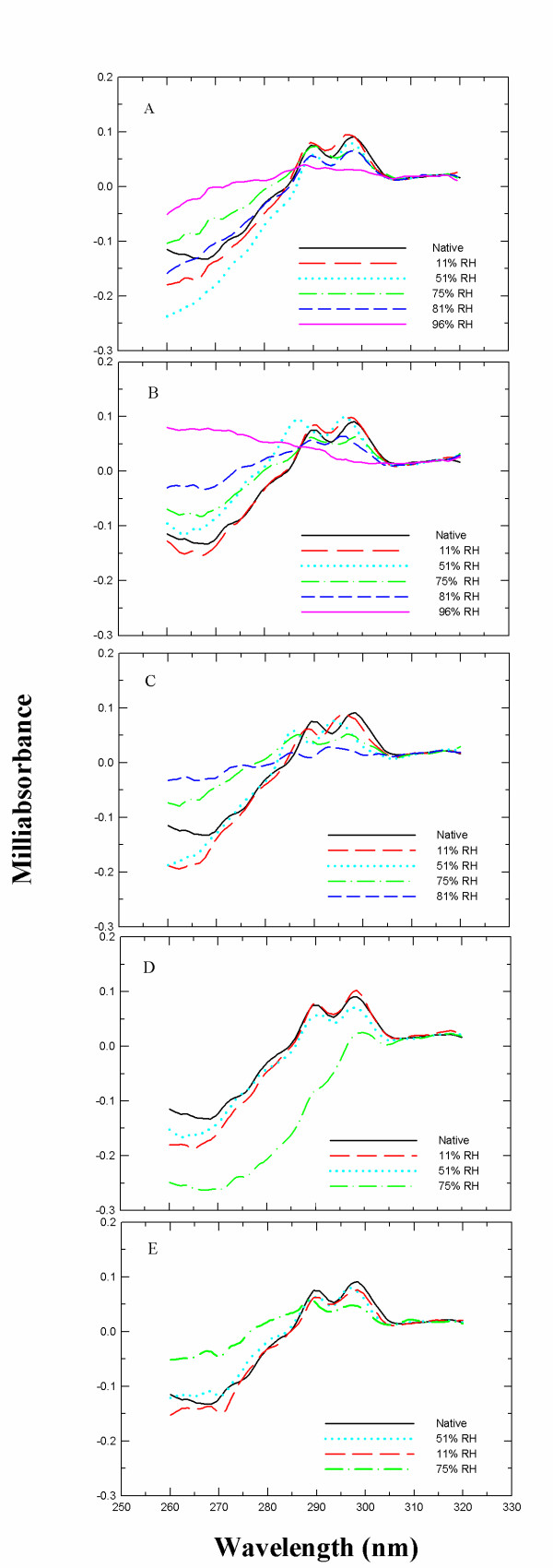
**Near-UV-CD spectra of α-CT dissolved in buffer after incubation at different RH and time intervals at 50°C**. (A) 24 h, (B) 48 h, (C) 72 h, (D) 96 h, and (E) 1 week incubation.

To determine protein structural changes induced during incubation, near-UV-CD spectroscopy was employed to examine the tertiary structure of the soluble fraction of the protein. After incubation the protein was redissolved in 10 mM phosphate buffer at pH 7.3 and 25°C and the solution was filtered to obtain the soluble fraction. The CD spectrum of α-CT displays two maxima at 288 and 297 nm (Figure 3) which completely disappear upon thermal unfolding. The data compiled demonstrate that structural changes were significant for almost all RH after a short period of storage (i.e., 24 h). This effect increased markedly at increasing RH, being most significant for the samples stored at 96% RH. After 1 week of incubation only the samples stored at 11% and 51% RH retained more than 60% of native structure. The data parallel those obtained with SEC- HPLC on monomer loss.

### Effect of Moisture on the Secondary Structure

Non-invasive FTIR microscopy was conducted to investigate the secondary structure of the samples during incubation using diamond windows [33] rather than pressing the powders into KBr pellets which is usually done to investigate the structure of lyophilized proteins [34,35]. FTIR analysis was performed in the amide III spectral region because water and water vapor have an insignificant contribution in this spectral area [34]. The amide III spectral region has the advantage that structural transitions can be monitored easily without spectral deconvolution because the absorptions of α-helix (ca. 1330-1290 cm^-1^), other, and β-sheet (ca. 1250-1215 cm^-1^) secondary structure are well separated [34]. First, we obtained FTIR spectra of the lyophilized powder using the usual KBr-pellet method [34] and by FTIR microscopy [33]. The spectra obtained were very similar and demonstrate that the method of FTIR spectral acquisition and sample preparation did not influence the result substantially in agreement with literature data [33].

Qualitative analysis of the spectra revealed that some spectral changes occurred in the amide III region after incubation of all powders under the RH conditions described. The shape of the spectra obtained even at 11% RH is somewhat different from those obtained without incubation. A slight increase in FTIR absorption is visible in the region corresponding to α-helix structure (ca. 1330-1290 cm^-1^), while β-sheet structure contributions to the spectra (ca. 1250-1215 cm^-1^) decreased. There is also more non- repetitive secondary structure present as indicated by the relative increase of the component around 1260 cm^-1^. We can conclude from this that some structural changes occur to αCT powder upon exposure to even low levels of moisture. Incubation of the samples for 96 h at different RH resulted in marked spectral changes characterized by an increase in the components around 1230 cm^-1 ^indicating an increase in the β-sheet content while the α-helix content suffered a drastic decrease even for the samples incubated at the lowest RH (11%). Quantitative analysis by calculation of the spectral correlation coefficient (SCC) also demonstrated that moisture sorption induced structural changes in αCT even at low RH (Table 2). A value of 1 demonstrates spectral and thus structural identity while lower values indicate spectral and thus structural changes. Again, as in all other tests, the magnitude of structural changes increased at increasing RH.

**Table 2:** Spectral correlation coefficients calculated from the amide III spectra of lyophilized α-CT after incubation at different RH.

Sample	**Structural correlation coefficient**^***a***^
11% RH 24 h	0.90 ± 0.03
72 h	0.88 ± 0.02
96 h	0.75 ± 0.05

51% RH 24 h	0.83 ± 0.00
72 h	0.85 ± 0.03
96 h	0.80 ± 0.05

96% RH 24 h	0.83 ± 0.05
72 h	0.83 ± 0.03
96 h	0.61 ± 0.04

^***a ***^A value of 1 demosntrates that the spectral are identical (and thus the secondary structure); values <1 show perturbations in the secondary structure.

### Structural Dynamics of Solid Proteins Exposed to Moisture

From the results presented thus far it is evident that structural changes occur to the solid protein formulations even when exposed to low RH levels and that structural changes become more pronounced at increasing RH levels. This leads to the hypothesis that moisture sorption increases structural dynamics in the lyophilized formulation.

Amide H/D exchange measurements have been used for over four decades to investigate protein dynamics. Despite of the compact folded structure of most proteins, there are solvent exposed amide bonds that exchange rapidly, but the amide bonds that are inaccessible to the solvent or are participating in stable hydrogen bonds must undergo temporary structural distortion to allow for hydrogen exchange and thus have significantly slower exchange rates. This decrease in the exchange rates of buried amide bonds in a folded protein makes H/D exchange an excellent and sensitive probe for monitoring protein conformational dynamics processes [23,25,36].

**Figure 4:**
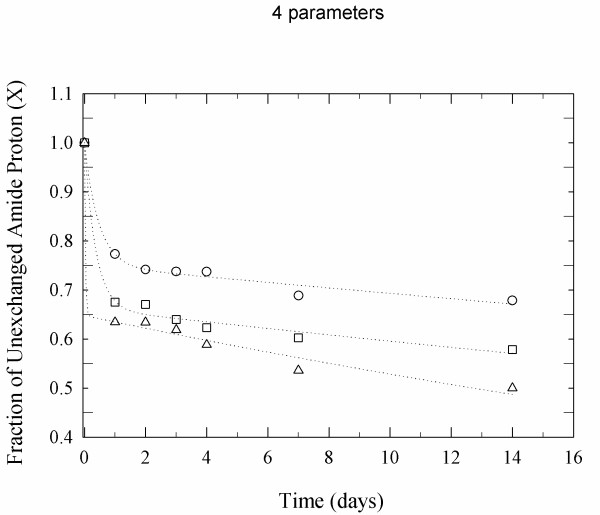
**H/D exchange plot of non-exchanged amide hydrogens of α-CT at different RH in D2O (pD 7.8)**. 11% RH (open circle), 51% RH 9 (open triangle), and 96% RH (open square).

In order to study the global structural dynamics, H/D exchange kinetics were measured by FTIR spectroscopy by following changes in the amplitude of the amide II band. The amount of amide hydrogen exchange (HX) was plotted in the form of HX decay plots where the fraction of unchanged amide hydrogen atoms (X) decreases over time due to the exchange process (Figure 4). Visual inspection of the Figure 4 demonstrates that at increasing RH level a decrease in the fraction of slowly exchanging amide bonds is observed. This demonstrates reduced structural rigidity of the enzyme at increasing RH values. A quantitative analysis of the exchange kinetics was attempted but led to insufficient fitting results because the fast exchange component could not be described appropriately.

### Residual Activity of α-Chymotrypsin after Incubation at Different RH

Activity of α-CT after incubation at different RH was determined using n- succinyl-Ala-Ala-Pro-Phe-p-nitroanilide as the substrate [23]. The activity of α-CT was reduced depending on the RH and the time the protein was exposed to it. Exposure to increased RH caused a more pronounced activity decrease (Table 3). The activity decrease parallels the loss of native-like structure observed by CD spectroscopy.

**Table 3:** Residual activity of α-CT after incubation at different RH

Residual Activity (%)					
**Time** **(d)**	**11% RH**	**51%RH**	**75% RH**	**81% RH**	**96% RH**

1	84 ± 2	75 ± 1	67 ± 2	37 ± 0.6	6 ± 0.4
2	82 ± 1	58 ± 2	40 ± 1	20 ± 0.4	2 ± 0.2
3	62 ± 2	58 ± 2	56 ± 2	8 ± 0.1	0.4 ± 0.1
4	75 ± 2	65 ± 1	49 ± 1	3 ± 1	n.d.
7	45 ± 3	40 ± 6	39 ± 5	n.d.	n.d.
14	39 ± 6	33 ± 5	23 ± 6	n.d.	n.d.
21	33 ± 2	28 ± 4	19 ± 8	n.d.	

Combining all data obtained thus far reveals the following likely scenario compatible with all observations: moisture sorption increases the structural dynamics of the protein which in turn enables structural changes leading to protein fragmentation, inactivation, and aggregation as the end result. All detrimental events increase at increasing RH during incubation and reach their maximum at the highest RH tested, namely 96% RH. Based on this we developed the following working-hypothesis: if it were possible to prevent water-protein interactions by shielding the protein surface and thus prevent an increase in structural dynamics, it should be possible to minimize or prevent the subsequent detrimental events. One method previously found to accomplish this goal is through the chemical glycosylation of the protein surface [21-24]. Solá *et al. *found through experimental and computational studies that chemically bound glycans can locally shield the protein surface from the interaction with the solvent (water) electrostatics thus reducing structural dynamics and increasing protein thermodynamic and colloidal stability [21,24]. These effects were mainly related to a reduction of solvent dielectric shielding effects within the proteins internal core [24].

### Stabilization of α-Chymotrypsin by Chemical Glycosylation

Based on the developed working hypothesis, we next performed chemical glycosylation using chemically-activated glycans as previously described by Solá *et al. *[23]. Glycans of differing length (500 Da lactose and 10 kDa dextran) were employed since it was previously found that for different instabilities both the amount and the length of glycosylation had impacts on stability. For example, while thermodynamics effects were found to depend only on the amount of glycosylation, colloidal stabilization effects were also found to depend on the size of the glycans.

Glycans were covalently attached to α-CT via the reactivity afforded by the surface lysine residues (ε-amine groups) towards a succinimidyl functionalized linker. Synthesis conditions were adjusted to achieve an average number of glycan molecules bound to the protein between 4 and 7 since maximum thermodynamic and colloidal stabilization effects in solution were previously reported for these constructs by Solá *et al. *[21-24]. Since the protein has 14 lysine residues, these modifications correspond to 30% and 50% of the total glycan content that can theoretically be attached.

**Figure 5:**
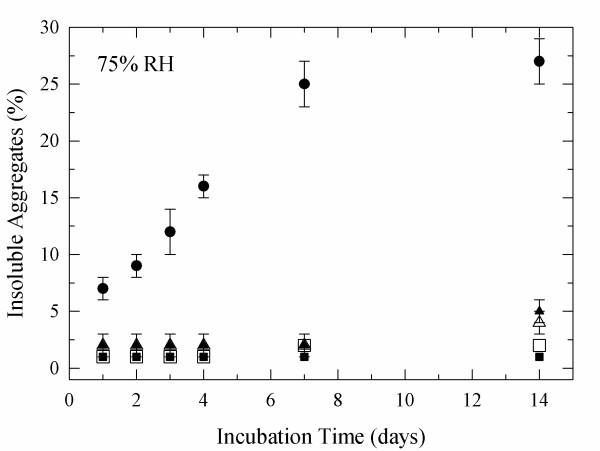
**Aggregation of glycosylated α-CT at 75% RH and 50°C**. Non-modified (solid circle), α-CT-lactose 4.5 ± 0.3 (open triangle), α-CT-lactose 7.1 ± 0.1 (solid triangle), α-CT-dextran 4.3 ± 0.3 (solid square), and α-CT-dextran 7.4 ± 0.1 (open square).

**Figure 6:**
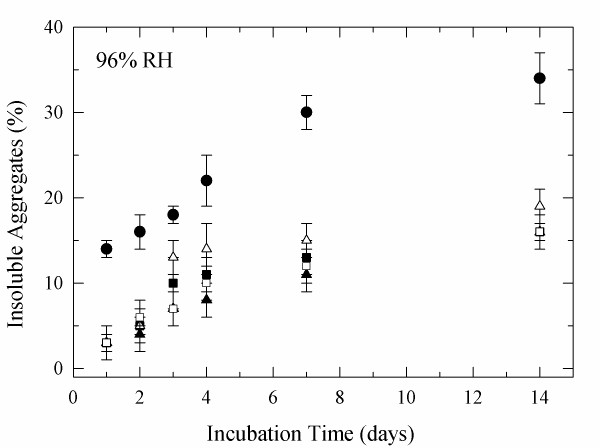
**Aggregation of glycosylated α-CT at 96% RH and 50°C**. unmodified (solid circle), α-CT-lactose 4.5 ± 0.3 (open triangle), α-CT-lactose 7.1 ± 0.1 (solid triangle), α-CT-dextran 4.3 ± 0.3 (solid square), and α-CT-dextran 7.4 ± 0.1 (open square).

To test the efficiency of the glycoconjugates, they were than incubated at 75% and 96% RH were most detrimental changes were observed. It was found that glycosylation was efficient in reducing the moisture-induced aggregation of α-CT (Figures 5, 6). It is evident that this effect was more pronounced at 75% RH (~25% reduction) than at 96% RH (~20% reduction). Overall both sugars were similarly efficient in preventing aggregation despite of the major difference in molecular weight. Similarly, glycosylation was also efficient in preventing activity loss (Tables 4 and 5).

**Table 4:** Residual Activity of α-CT modified with dextran after incubation at different RH.

	75% RH			96% RH		
**Time** **(d)**	**a-CT**	**a-CT Dextran** **4.3 ± 0.3**^***a***^	**a-CT Dextran** **7.4 ± 0.1**^***a***^	**a-CT**	**a-CT Dextran** **4.3 ± 0.3**^***a***^	**a-CT Dextran 7.4 ± 0.1**^***a***^

1	67 ± 2	84 ± 2	84 ± 0	6 ± 1	23 ± 2	51 ± 2
2	40 ± 1	70 ± 0	83 ± 3	2 ± 1	19 ± 2	38 ± 2
3	56 ± 2	65 ± 1	64 ± 2	0.4 ± 0.1	12 ± 2	25 ± 2
4	49 ± 1	59 ± 2	62 ± 2	no data	5 ± 2	17 ± 0
7	39 ± 5	66 ± 2	70 ± 6	no data	2 ± 2	8 ± 2
14	23 ± 6	54 ± 2	70 ± 2	no data	1 ± 2	12 ± 2

^***a***^Molar ratio of dextran-to-α-CT

**Table 5:** Residual Activity of α-CT modified with lactose after incubation at different RH.

	75% RH			96% RH		
**Time** **(d)**	**a-CT**	**a-CT Lactose** **4.5 ± 0.3**^***a***^	**a-CT Lactose** **7.1 ± 0.1**^***a***^	**a-CT**	**a-CT Lactose** **4.5 ± 0.3**^***a***^	**a-CT Lactose 7.1 ± 0.1**^***a***^

1	67 ± 2	100 ± 2	99 ± 3	6 ± 1	45 ± 2	59 ± 1
2	40 ± 1	105 ± 2	83 ± 3	2 ± 1	39 ± 2	51 ± 1
3	56 ± 2	90 ± 3	72 ± 2	0.4 ± 0.1	35 ± 1	26 ± 1
4	49 ± 1	74 ± 2	72 ± 1	no data	16 ± 1	22 ± 1
7	39 ± 5	76 ± 2	75 ± 2	no data	10 ± 1	11 ± 1
14	23 ± 6	75 ± 2	62 ± 2	no data	3 ± 2	5 ± 2

^***a***^Molar ratio of lactose-to-α-CT

Since it is evident from the results that stability was imparted regardless of the size of the glycan attached to the protein, stability improvements were probably not due to the glycans working as molecular spacers preventing aggregation by keeping (unfolded) protein molecules at a distance. In contrast to liquid storage stability under accelerated conditions in which dextran conjugates were much more efficiently protected from aggregation than lactose conjugates [22], no difference was found in the improvement of solid-phase storage stability herein.

However, glycans interact with the protein surface and this interaction is limited to a small portion of the glycan molecule close to the protein surface [24]. The glycan- protein interaction leads to a shielding of protein surface area from water and thus the protein dynamics and structure becomes more conformationally restricted [22,24]. The most likely mechanism by which glycans improve the solid-state stability of lyophilized α-CT involves this mechanism. In this work, we have presented evidence to support that moisture sorption leads to an increase in protein structural dynamics which causes structural changes of the protein in the lyophilized state. These structural changes then trigger partial/complete unfolding and aggregation of the protein. Reduction in the structural dynamics by glycosylation should therefore reduce these deleterious pathways.

## Conclusions

In this work it is demonstrated that moisture causes increased structural dynamics, structural changes, aggregation, and inactivation of α-CT in the solid-state. In general, the higher the RH was during incubation, the more pronounced these detrimental events were. Moreover, it was demonstrated that chemical glycosylation with lactose and dextran reduced and/or prevent these deleterious processes to occur in α-CT in the solid state. These results provide experimental evidence which suggest that the use of chemically attached glycans could be very useful in the development of solid-phase protein formulations to improve their stability during long-term storage even under accelerated conditions. The exact mechanism is at present not clear and theoretical studies might be needed, but water exclusion from the protein surface causing rigidification should be among the most favorite hypothesis.

## Methods

### Enzymes and reagents

α-CT (EC 3.4.21.1, type II from bovine pancreas) and Dextran (10 kDa) were purchased from Sigma-Aldrich (St. Louis, MO); mono-(lactosylamido)-mono- (succinimidyl) suberate (SS-mLAC); 2,4,6-trinitrobenzene sulfonic acid (TNBSA) were from Pierce (Rockford, IL) and succinyl-Ala-Ala-Pro-Phe-p-nitroanilide was from Bachem (King of Prussia, PA). All other chemicals were of analytical grade or purer and were obtained fromvarious commercial suppliers.

### Preparation of α-Chymotrypsin Samples

α-CT was dissolved in deionized water (0.5 mg/mL, pH 7.3). These samples were frozen in liquid N_2 _and lyophilized for 48 h using a Labcono FreeZone 6L freeze drier at a condenser temperature of -45°C and a pressure of <60 μm of Hg. The final moisture content of the lyophilized powders was 5.4 ± 1.5% (w/w) as determined by Karl Fisher titration with a Metrohm 831 KF Coulometer. After lyophilization and redissolving the samples, no change in pH was noted.

### Synthesis of Monofunctionally Activated Dextrans

Synthesis of mono-(dextranamido)-mono-(succinimidyl)suberate (10 kDa) (SS- mDEX) was carried out as previously described by Solá *et al. *[23] In brief, dextran (10 kDa) (10.0 g, 1.0 mmol) and ammonium carbonate (2.0 g, 21.74 mmol) in nanopure water (50 mL) were stirred at 10°C for 5 days. Afterwards ammonium carbonate was removed by dialysis and the solution was lyophilized to afford 1-amino-dextran (10 kDa). To achieve dextran succinnylation, 1-hydroxy benzotriazole (0.675 g, 5.0 mmol) and disuccinimidyl suberate (1.841 g, 5.0 mmol) were dissolved in DMSO (60 mL) and heated to 80°C for 5 min. After cooling, 1-amino-dextran (10 kDa) (5.0 g, 0.5 mmol) was added and the reaction maintained at 20°C for 24 h. The product was precipitated by addition of CH_2_Cl_2 _(200 mL) followed by centrifugation (1,500 rpm) at 48°C for 15 min. The resulting white precipitate was lyophilized to afford SS-mDEX in quantitative yield. Reaction products were characterized by colorimetric ninhydrin and orcinol tests [22,23].

### Synthesis of α-CT Glycoconjugates

Covalent modification of α-CT with lactose and dextran conjugates was performed by chemical glycosylation with SS-mLAC (500 Da) and SS-mDEX (10 kDa) as described by Solá *et al. *[23]. To achieve protein conjugates with varying glycan molar content different amounts of SS-mLAC and SS-mDEX were added to α-CT solution to achieve molar ratios of 4.5 and 7.1 mol of reagent per mol of protein in 0.1 M borate buffer, pH 9.0. Reaction mixtures were stirred at 4°C for 2 h. Unreacted glycans and buffer salts were removed by dialyzing against deionized water. The glycoconjugates were subsequently lyophilized and stored at -20°C until use. The degree of protein modification was determined by colorimetric titration of unreacted amino groups with TNBSA [23].

### Moisture-Induced Aggregation

To study the effect of moisture on α-CT and glycoconjugate solid-phase stability, we exposed the lyophilized powder (0.5 mg) in an Eppendorf tube to accelerated storage conditions, i.e., controlled humidity and high temperature. Incubation of the lyophilized powders at constant levels of RH was performed by vapor equilibration in dessicators over salt slush: LiCl (11% RH), NaBr (51% RH), NaCl (75% RH), MgCl_2 _(81% RH), and K_2_SO_4 _(96% RH) [13]. The desiccators were kept in a LabLine incubator at a constant temperature of 50°C. These chambers were left for 48 h closed to reach equilibrium. Following the desired length of time, the incubated protein sample was removed and 1 mL of phosphate buffered saline (PBS, 10 mM pH 7.3) and a magnetic stirrer was added followed by 2 h of gentle stirring to ensure dissolving the soluble fraction of the sample. Soluble and aggregated protein were separated by centrifugation in a microcentrifuge at 9000 rpm for 10 min. To determine the amount of aggregated protein 1 mL of 6 M urea was added to the pellet and left stirring overnight. The protein concentration in the resulting clear solution was determined by absorption measurement at 280 nm. The experiments were performed in triplicate and the results averaged. Error bars in the figures are the calculated standard deviations.

### Determination of Enzyme Activity

The enzymatic activity of α-CT and α-CT glycoconjugates was determined by measuring the rate of hydrolysis of the substrate succinyl-Ala-Ala-Pro-Phe-p- nitroanilide [23]. Product formation (p-nitroaniline) was followed by measuring the formation of p-nitroanilide at 410 nm (ε410 = 8.8 mM-1 cm-1). Reactions were carried out in 10 mM potassium phosphate buffer (pH 7.1, 25°C). All reactions were started by addition of 60 μL of enzyme ([E]_0 _= 0.8 μM) to 240 μL substrate solution ([S]_0 _= 0.8 mM) in a final volume of 1 mL. Initial velocities were obtained from the linear portion of plots of the concentration of product formed *vs*. time.

### Measurement of α-CT Degradation

Lyophilized samples of α-CT (0.5 mg) were prepared at pH 5 and stored in the humidity chambers at a constant temperature of 50°C under accelerated storage conditions. These samples were removed at various times and dissolved in 10 mM PBS at pH 7.3, and filtered through a Millex-GV filter (Millipore) of 0.22-μm pore size to remove insoluble materials, and then subjected to SEC-HPLC using a Perkin Elmer ISS 200 HPLC and an Applied Biosystems 759A detector. The column used was a Tosoh Bioscience TSKgel SuperSW3000 (4.6 mm × 30 cm, 4.6 μm) maintained at 25°C. Elution was performed using 50 mM NaH_2_PO_4 _containing 100 mM NaCl, pH 7.0, at a flow rate of 0.5 mL/min. The detection wavelength was 280 nm (see Figure 1 of the Supporting Information). The soluble fraction was determined from the monomer peak area before and after different incubation.

### Circular Dichroism (CD) Measurements

Near-UV circular dichroism measurements were acquired with an Olis DSM-10 UV-Vis CD spectrophotometer. The protein concentration was adjusted to 0.6 mg/ml in 10 mM potassium phosphate buffer at pH 7.3 and 25°C. Spectra were recorded from 260 to 320 nm using a 1.0 cm path length quartz cell. Each spectrum was obtained by averaging six scans at 2 nm resolution. Solvent reference spectra were digitally subtracted from protein CD spectra.

### FTIR Amide H/D Exchange Kinetics

#### Protein Preparation for H/D Exchange Experiments

Lyophilized α-CT (2 mg) was hydrated by vapor equilibration using saturated solutions of aforementioned salts in D_2_O. To reduce the water content of the salts, the salts were dried in an oven for 2 h at 120°C. The samples were incubated for different time intervals at different RH in D_2_O at 50°C. Once the period of incubation had passed the samples were lyophilized for 24 h. The protein powder and 200 mg of KBr were mixed and ground to a fine powder with a mortar and pestle. The resulting powder was pressed into a KBr disk.

#### H/D Exchange Assay

Amide H/D exchange FTIR spectra were measured using a Nicolet NEXUS 470 infrared spectrometer equipped with a thermally controlled sample cell (Spectra-Tech Inc., Shelton, CT). For all spectra 56 scan at 4 cm-1 spectral resolution were collected and digitally averaged.

#### Processing of H/D Exchange Data

H/D exchange spectra from experiments were processed for quantitative analysis in form of hydrogen exchange decay plots. The fraction of unexchanged amide hydrogen atoms (X) was defined as:

(1a) $$X=\frac{w(t)-w(\infty )}{w(0)-w(\infty )}$$

where w(t) is the ratio of the amide II (1550 cm-^1^) and amide I (1637.5 cm^-1^) absorbencies corrected with the baseline absorbance (1789 cm^-1^) at time t, w(0) is the amide II/amide I ratio of the non-deuterated protein, and w(∞) is the amide II/amide I ratio for the fully deuterated protein.

### Fourier Transform Infrared (FTIR) Microscopy

FTIR-microscopy studies were performed using a Nicolet Magna-IR 870 bench [1] and a Thermo Spectra-Tech Continuum microscope using a diamond cell (Thermo Spectra-Tech, Diamond windows) to avoid exposing the moist protein powders to the high pressure encountered during the formation of KBr pellets usually employed in such studies [13,33]. Spectra were recorded in the transmission mode at 4 cm^-1 ^resolution and 256 scans were averaged to obtain one spectra. To avoid artifacts by water vapor and sorbed water, the amide III region (1220-1330 cm^-1^) was used for the analysis of protein secondary structure since the absorbance of water is negligible in this spectral region [34]. It should be noted that the use of the amide III region to investigate the secondary structure of hydrated and dehydrated proteins has been validated by simultaneous analysis of the amide I and III region [1,37,38].

### Calculation of the Spectral Correlation Coefficient

Spectral changes caused by moisture were quantified by calculating the spectral correlation coefficient (SCC) [34,38]. The SCC value reflects differences of two spectra: for identical ones it is 1, for those with nothing in common it is 0. The second-derivative spectra in the amide III region (1220-1330 cm-^1^) were calculated and saved for the spectral range on identical wavenumber scales and with identical data spacing. The data for the reference spectrum (in this case the lyophilized powder measured by FT-IR microscopy) and the spectrum with the varied condition (e.g., RH and incubation time) were imported into the program SigmaPlot and the correlation coefficient was calculated [34,38].

### Determination of Water Sorption Isotherms

Measurements of protein-bound water were conducted by Karl Fisher titration using hydranal solvent with a Metrohm 831 KF coulometer. This method has been routinely used to measure the water content of protein powders. Samples (0.5 mg) each were stored at various RH. Following storage, 1.0 mL of anhydrous dimethylsulfoxide was added to each sample. This solution was then sonicated for 30 sec and injected into the coulometer. All water contents are reported as percentages (w/w). Equilibrium uptake of water by the lyophilized powder was achieved and no additional water was absorbed after a maximum of 8 h at 50°C for all RH.

### Statistical Methods

All statistical analyses were done in Minitab 14 (Minitab Inc., State College, PA). Statistical analysis of the effect of the water content (11%, 51%, 75%, 81% and 96% RH) on the quantity of insoluble aggregates formed after 24 h, 48 h, 72 h, 96 h, 1 week, and 2 weeks was performed using the Kruskal-Wallis test and the individual differences between the various samples. Post-hoc comparisons of the means of individual groups were performed using Dunn's test. Statistical significance was accepted at the p ≤ 0.05 level. All experiments were performed at least in triplicate, the results averaged, and the standard deviations calculated.

## Authors' contributions

GMFF carried out the aggregation, CD, HPLC and H/D studies, contributed to the experimental strategy, analyzed data, and drafted the manuscript. MP carried out the chemical glycosylation modification and enzyme kinetics studies, analyzed data, and helped draft the manuscript. MA helped in the aggregation, glycosylation, and enzyme kinetics studies. RS helped designing some of the studies and revised the manuscript. KG conceived the study, participated in its design and coordination, and finalized the manuscript. All authors read and approved the final manuscript.

## Supplementary Material

Additional file 1 **Water sorption isotherms of α-CT incubated at different RH at 50°C measured by Karl Fisher coulometry**. BET water sorption isotherms of α-CT demonstrating water sorption equilibration after incubation at different RH Click here for file

Additional file 2 **SEC-HPLC chromatogram of α-CT incubated at 96% RH and 50°C. α-CT was dissolved at pH 5**. Chromatograms depicts the monomer loss of α-CT after incubation at 96% RH Click here for file
