# Supplementary figures and images for: Moisture-induced solid state instabilities in α-chymotrypsin and their reduction through chemical glycosylation
Source: BMC Biotechnol. 2010 Aug 9;10:57. doi: 10.1186/1472-6750-10-57 (PMC2924255; doi:10.1186/1472-6750-10-57)

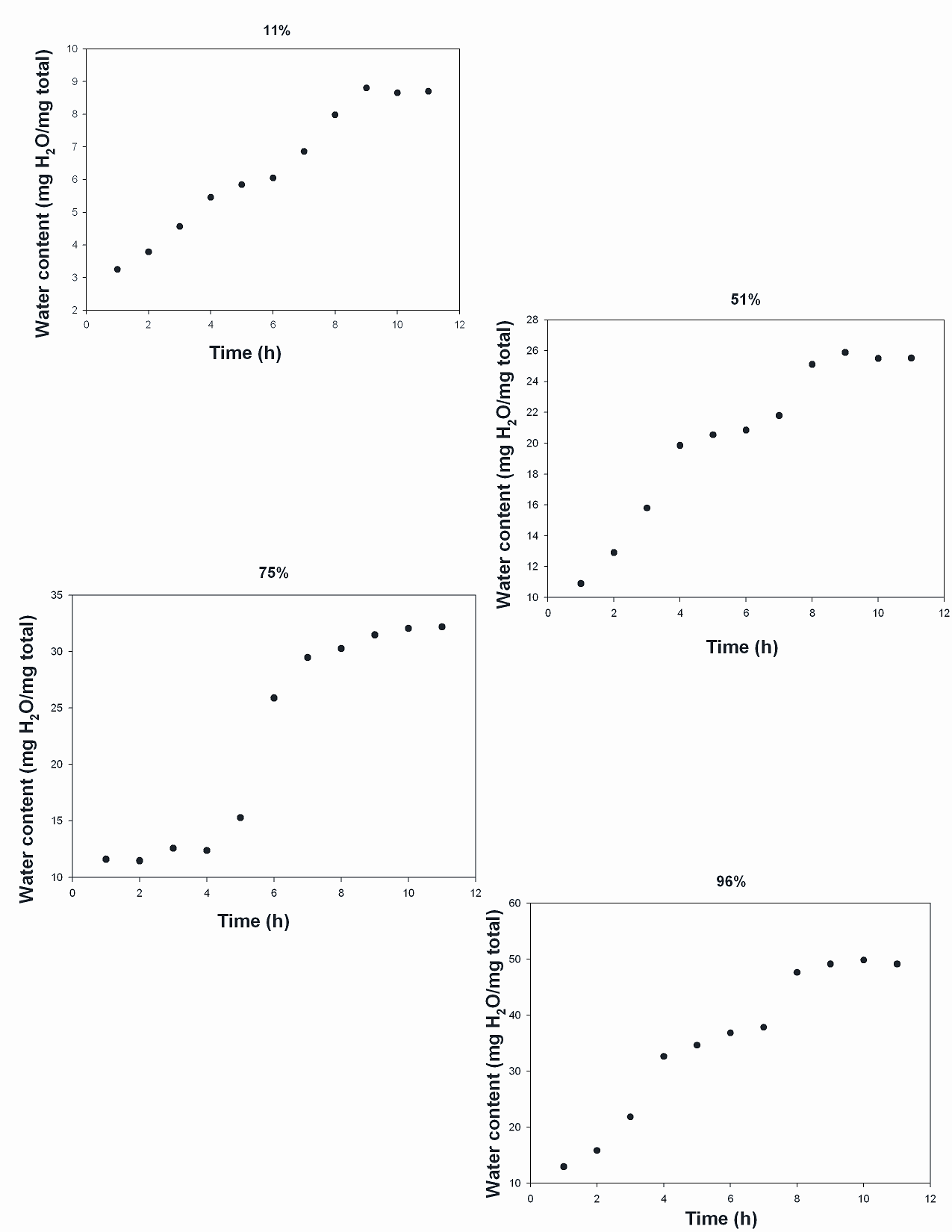

Supplement: Additional file 1 — Water sorption isotherms of α-CT incubated at different RH at 50°C measured by Karl Fisher coulometry. BET water sorption isotherms of α-CT demonstrating water sorption equilibration after incubation at different RH [file 1472-6750-10-57-S1.TIFF]
